# Analysis of Doline Microtopography in Karst Mountainous Terrain Using UAV LiDAR: A Case Study of ‘Gulneomjae’ in Mungyeong City, South Korea

**DOI:** 10.3390/s25144350

**Published:** 2025-07-11

**Authors:** Juneseok Kim, Ilyoung Hong

**Affiliations:** Department of Drone & GIS Engineering, University of Namseoul, Cheonan City 31020, Republic of Korea; junesoek.kim@nsu.ac.kr

**Keywords:** UAV, LiDAR, doline, DEM, karst, microtopography

## Abstract

This study utilizes UAV-based LiDAR to analyze doline microtopography within a karst mountainous terrain. The study area, ‘Gulneomjae’ in Mungyeong City, South Korea, features steep slopes, limited accessibility, and abundant vegetation—conditions that traditionally hinder accurate topographic surveying. UAV LiDAR data were acquired using the DJI Matrice 300 RTK equipped with a Zenmuse L2 sensor, enabling high-density point cloud generation (98 points/m^2^). The point clouds were processed to remove non-ground points and generate a 0.25 m resolution DEM using TIN interpolation. A total of seven dolines were detected and delineated, and their morphometric characteristics—including area, perimeter, major and minor axes, and elevation—were analyzed. These results were compared with a 1:5000-scale DEM derived from the 2013 National Basic Map. Visual and numerical comparisons highlighted significant improvements in spatial resolution and feature delineation using UAV LiDAR. Although the 1:5000-scale DEM enables general doline detection, UAV LiDAR facilitates more precise boundary extraction and morphometric analysis. The study demonstrates the effectiveness of UAV LiDAR for detailed topographic mapping in complex karst terrains and offers a foundation for future automated classification and temporal change analysis.

## 1. Introduction

Karst refers to a terrain formed over carbonate rocks such as limestone and manifests in various forms depending on its scale, including dolines, uvalas, and poljes [1,2]. Among these, dolines are the most representative karst landforms, and a quantitative morphological analysis is essential to understand their origins, developmental processes, land use and pollution patterns, and subsidence characteristics [3,4].

Traditional analyses of dolines have primarily relied on field surveys, digital topographic maps, and aerial photography, interpreting geomorphological features through slope measurements, cross-sectional profiling, and soil and rock analysis [5]. However, these methods are limited in terms of resolution and accuracy, particularly in areas with dense vegetation or steep slopes, where detailed terrain recognition is challenging [6,7].

While technologies such as Aerial Laser Scanning (ALS) and LiDAR, which provide high-resolution terrain data, have been recognized as promising solutions to these limitations, their high costs and operational constraints have restricted their applicability for repeated surveys or analyses over small areas [8]. Recently, UAV-based Structure from Motion (SfM) techniques have emerged as a cost-effective alternative for generating high-resolution data; however, they still face limitations in accurately extracting terrain features beneath vegetation cover [5,9].

As an alternative to address these challenges, UAV LiDAR technology has emerged as a promising solution [10]. UAV LiDAR can penetrate vegetation to acquire highly precise elevation data of the ground surface and is capable of generating high-density point cloud datasets, making it particularly well-suited for microtopographic analysis [1,11,12,13]. In particular, it can be effectively applied to doline studies where the precise extraction of boundaries is crucial [3,4].

With recent advances in sensor miniaturization and cost reduction, UAV LiDAR applications have expanded across various fields, including cartography, urban planning, forest management, and archaeology [14,15,16]. Its utility is especially high in mountainous terrains where ground access is difficult, allowing for the rapid and precise collection of spatial data. Recent studies have increasingly applied high-resolution LiDAR data for doline morphometry in diverse karst environments [17], highlighting its utility in capturing detailed terrain features.

This study aims to generate high-resolution Digital Elevation Models (DEMs) using UAV LiDAR for dolines distributed across the ’Gulneomjae’ karst mountainous area in Bugok-ri, Hogye-myeon, Mungyeong City, Gyeongsangbuk-do, South Korea. Based on these DEMs, quantitative geomorphic indicators such as perimeter, depth, and volume of the dolines were analyzed. Additionally, the precision and practical applicability of UAV LiDAR were assessed by comparing the results with existing analyses based on digital topographic maps and field surveys conducted in a 2013 study [18]. This research empirically demonstrates the effectiveness of UAV LiDAR-based microtopographic analysis for the precise detection and classification of complex landforms such as dolines, and it further suggests the potential for broader applications in diverse geomorphic environments.

## 2. Related Work

The integration of UAV and LiDAR technologies has rapidly established itself as an efficient means of acquiring high-resolution topographic data and is being utilized in a variety of fields such as terrain analysis, ecosystem monitoring, and precision agriculture [19,20]. UAV LiDAR, in particular, offers superior point density and vertical accuracy compared to airborne LiDAR or Structure from Motion (SfM) methods, providing distinct advantages for small-scale and microtopographic change analysis [15,21,22].

These characteristics make UAV LiDAR particularly effective for quantitative analysis in regions with complex micro topographies, such as karst terrains. Ref. [1] employed UAV LiDAR to conduct detailed analyses of karst features across various scales in Brazil’s semi-arid regions, while ref. [2] utilized LiDAR data to quantitatively classify conical karst features in temperate regions. Ref. [3] analyzed the correlation between forest understory vegetation and doline shapes, and ref. [4] proposed an algorithm for automatically extracting sinkholes from LiDAR-based Digital Elevation Models (DEMs).

Studies focusing on the generation and accuracy assessment of UAV LiDAR-based DEMs are also abundant. Refs. [5,6] compared the precision of UAV photogrammetry and LiDAR-based DEMs, while ref. [23] analyzed the accuracy of Digital Terrain Models (DTMs) generated from both data sources. Additionally, ref. [15] proposed an accuracy evaluation technique utilizing the DJI ZENMUSE L1 sensor.

Beyond terrain analysis, UAV LiDAR has found widespread application in ecological fields such as vegetation and biomass estimation, and biodiversity monitoring. Ref. [11] demonstrated the effectiveness of UAV LiDAR in estimating biomass in grasslands, while Refs. [24,25] utilized it to analyze structural indicators of shrubs and trees. Ref. [26] analyzed the impact of LiDAR point density on biomass estimation accuracy, and ref. [12] assessed the effect of LiDAR data loss on structural estimation in grassland environments.

Additionally, UAV LiDAR has been applied in various ecological and agricultural contexts, including vegetation stratification [27,28], mangrove ecosystem classification [29], and crop identification and growth estimation [30]. In terms of data fusion, systems integrating GNSS/INS-based camera-LiDAR [31], combinations of hyperspectral imagery and LiDAR [32], and machine learning-based biomass prediction [33] have garnered attention.

UAV LiDAR has also proven effective in special terrain analyses. Ref. [7] applied it to coastal karst analysis, ref. [34] combined drone imagery with cave networks to analyze geological structures, and ref. [35] used airborne LiDAR to detect cave entrances beneath forests. Ref. [36] reported on the classification of restored tropical forest types using UAV LiDAR.

Finally, the potential applications of UAV LiDAR in urban flood modeling and infrastructure monitoring are expanding [16,37]. These diverse preceding studies underscore that UAV LiDAR has firmly established itself as a key tool for high-resolution microtopographic analysis and quantitative terrain interpretation. Building on this technological trend, this study aims to apply UAV LiDAR to the precise morphological analysis of dolines in karst mountainous regions.

## 3. Materials and Methods

This study focused on the karst terrain of the mountainous region in Bugok-ri, Hogye-myeon, Mungyeong City, South Korea, and conducted high-resolution microtopographic analysis using UAV LiDAR technology. To achieve this, the research process was systematically structured, including an overview of the characteristics of the study area, the equipment and data collection conditions, data processing procedures, and the comparison methodology with existing DEM based on traditional topographic maps.

### 3.1. Study Site

**Figure 1:**
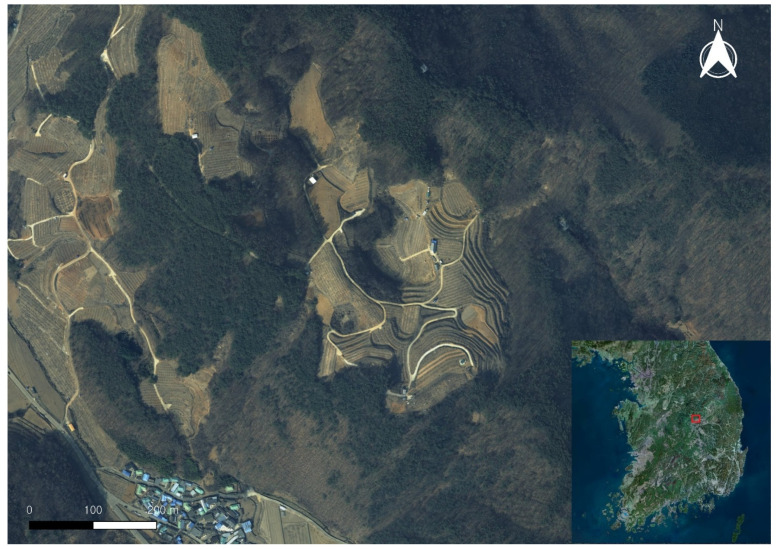
The site of the doline topography study. (The red square indicates the location of Gulneomjae in the Republic of Korea.)

Figure 1 shows the Gulneomjae area in Bugok-ri, Hogye-myeon, Mungyeong City, which is the study area for this research. The Gulneomjae region is a karst landscape underlain by limestone, characterized by the distribution of dolines, uvalas, and poljes of various sizes and shapes. The area is composed of steep slopes and dense vegetation, making it difficult to obtain precise topographic information using traditional terrain analysis methods. Therefore, this study applied UAV LiDAR technology to analyze the quantitative characteristics and microtopographic structures of the dolines distributed in the region.

### 3.2. UAV LiDAR Data Collection

The UAV-based airborne LiDAR survey was conducted on 23 September 2024, using the DJI Matrice 300 RTK unmanned aerial vehicle to acquire precise topographic data. This UAV is equipped with Real-Time Kinematic (RTK) positioning capabilities, which allows for the acquisition of high-precision location data without the need for separate Ground Control Points (GCPs).

The sensor onboard is the DJI Zenmuse L2 LiDAR system, which supports the enhanced Penta Return mode compared to the previous L1 sensor, offering superior surface detection capability even in densely vegetated areas. The scanning mode was set to Repeated Scan, which, while narrowing the scanning range, ensures higher precision and is thus suitable for high-resolution topographic analysis.

**Figure 2:**
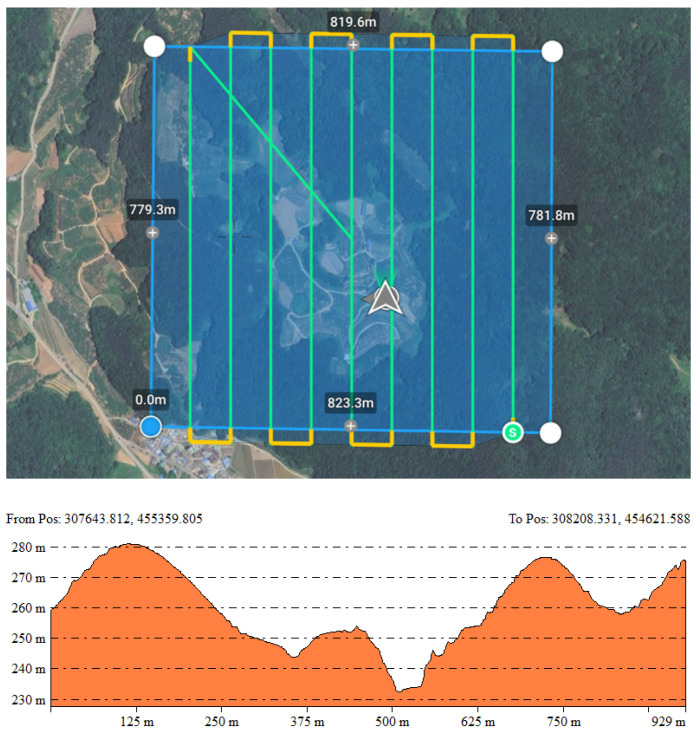
Flight route of UAV over study site and elevation profile of study site.

The flight was conducted considering the terrain characteristics of the study area (altitude range approximately 237–277 m), with the flight altitude set to 150 m to avoid surrounding obstacles such as power lines (Figure 2). The UAV’s speed was set to 10 m/s, and the camera angle was fixed at −90° (vertical to the ground) to maximize the vertical accuracy of the LiDAR point cloud (See Table 1).

During the survey, RGB imagery was collected with a front overlap of 70% and a side overlap of 61%, while the LiDAR data was acquired with a side overlap of 50%. The UAV conducted the survey over a total area of 0.64 km^2^, and the collected LiDAR point cloud exhibited an average point density of 98 points per square meter. This setup enabled the acquisition of precision exceeding that of conventional aerial surveying standards, providing the foundational data for subsequent DEM generation and doline morphological analysis.

### 3.3. Data Processing and DEM Generation

**Figure 3:**
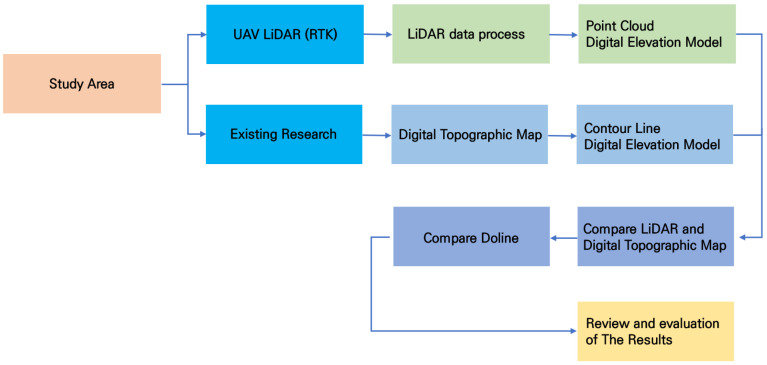
Research flow chart.

Figure 3 illustrates the overall flow of the research. The process of the study consisted of data collection for the study area, data processing, creation of comparison DEMs, comparison with previous research results, and analysis of the detailed topography of the dolines.

The collected LiDAR data underwent preprocessing, alignment, and filtering using DJI Terra software version is 4.2.5, ultimately resulting in a DEM with a resolution of 0.25 m. The point cloud was classified according to attributes such as reflectivity, height, return number, and point type. Only the ground points were selected for DEM construction. The DEM was based on the EPSG:5187 (Eastern Origin) coordinate system, with vertical accuracy ensured through the application of the KNGeoid18 geoid model, securing both spatial and vertical alignment. The generated high-resolution DEM was subsequently used as the foundational data for key analysis steps, including doline location detection, shape analysis, and quantitative comparisons.

Table 2 presents the accuracy parameter values related to IMU trajectory errors during the LiDAR scanning process, while Table 3 summarizes the parameters associated with the point cloud density generated after LiDAR scanning, organized by map scale.

For comparison with the UAV LiDAR-based DEM, we separately generated a digital elevation model (DEM) from the 1:5000-scale National Basic Map V2.0, provided by the National Land Information Platform. Contour lines were extracted, and a DEM was constructed using the Triangulated Irregular Network (TIN) interpolation method. A shaded relief map and a contour map were then produced within the same spatial extent to facilitate visual and quantitative comparisons. Although the resolution and precision of this national basic map-derived DEM are comparatively lower, it provides a valuable reference for analyzing historical topographic conditions and temporal changes.

## 4. Results

### 4.1. Accuracy and Representation of DEMs

**Figure 4:**
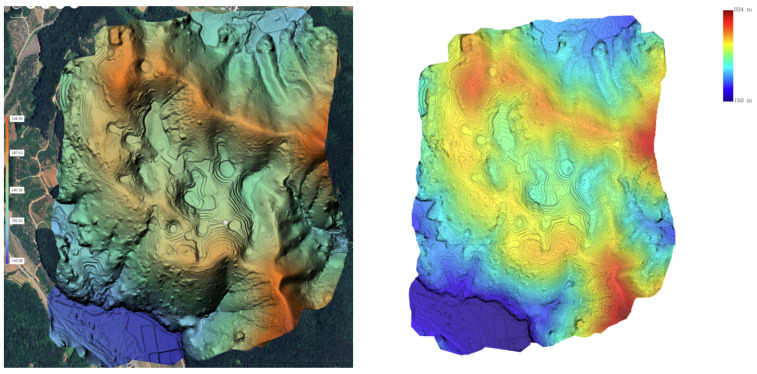
DEM and contour line acquired with UAV LiDAR.

The UAV LiDAR-based DEM generated in this study, with a resolution of 0.25 m and an average point density of 98 pts/m^2^, allowed for highly detailed topographic representation. When compared to the DEM derived from the national basic map, the UAV LiDAR-based DEM provided a clearer visualization of fine topographic features, such as the boundaries, shapes, and depths of the dolines. Particularly in areas with dense vegetation, the UAV LiDAR effectively captured the ground surface, overcoming the resolution limitations of the traditional contour-based DEM (see Figure 4).

**Figure 5:**
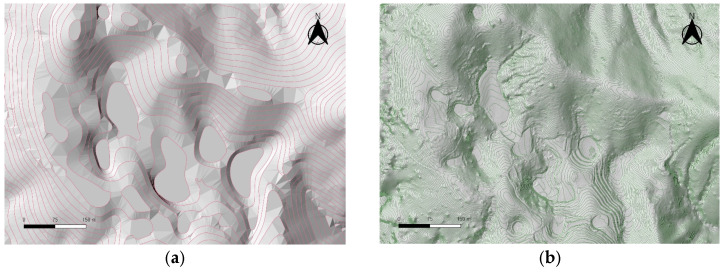
Shaded relief map and 5 m contour lines generated from 1:500 digital topographic map (**a**); shaded relief map and 0.25 m contour lines generated from UAV LiDAR (**b**).

Visual comparison using shaded relief maps and contour maps also demonstrated that the UAV LiDAR-based DEM provided high-quality precision and more accurately represented the actual topographic forms. In contrast, the DEM based on the national map, with a contour interval of 5 m, simplified the topographic complexity, making it difficult to accurately reproduce the actual shape of the dolines (see Figure 5).

### 4.2. Doline Detection and Spatial Comparison

**Figure 6:**
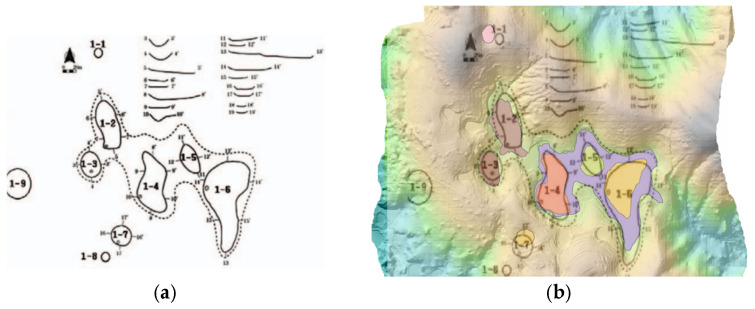
Comparison of doline location. Field survey results from 2013 (**a**) overlayed with UAV LiDAR-based detection results (**b**).

**Figure 7:**
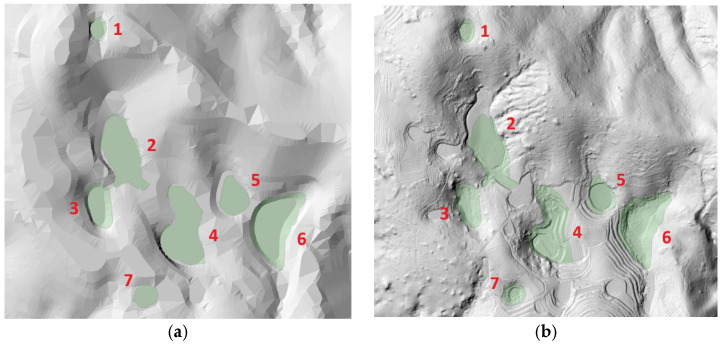
Comparison of each doline (ID: 1–7) between contour-based mapping (**a**); UAV LiDAR detection (**b**) (the green polygon is the results of a survey conducted in 2013).

The comparison of UAV LiDAR-based detection results with a previous field survey conducted in 2013 [18], which utilized a 1:5000-scale national basic digital elevation model, revealed that seven out of the nine dolines reported at that time were clearly identified in the present study. The DEM from the 2013 study was constructed based on contour data extracted from the National Basic Map V2.0, allowing for a historical comparison despite its lower spatial resolution. The remaining two dolines may have been altered due to external factors such as cultivation, erosion, and terrain changes, or it is possible that detection errors occurred due to the low resolution of the existing data (see Figure 6 and Figure 7).

**Figure 8:**
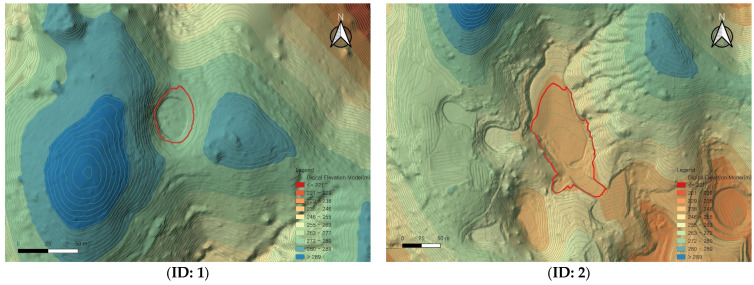

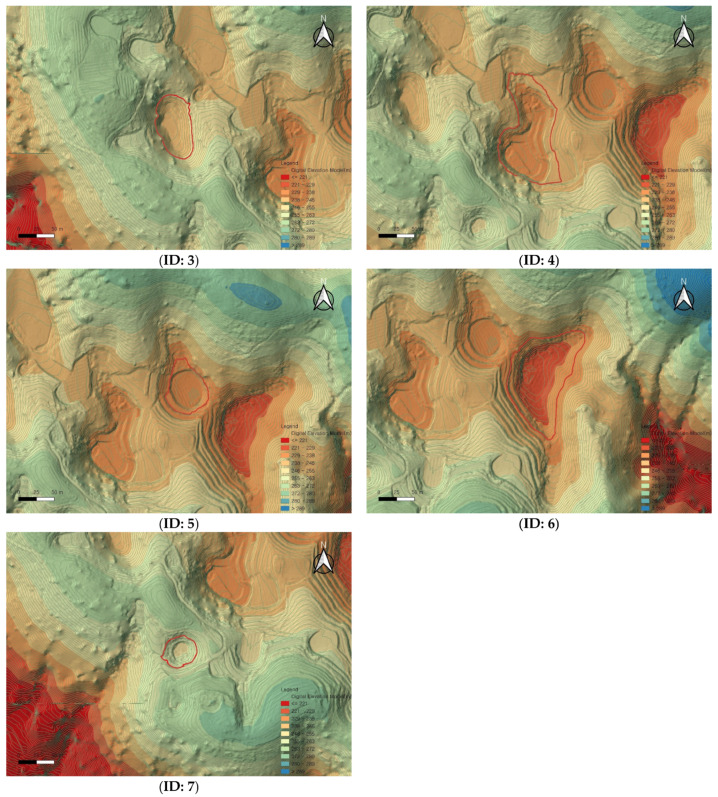
Location of each doline (**ID: 1**–**ID: 7**) on the UAV LiDAR-based DEM and contour lines (red outline indicates 2013 survey).

When comparing the doline boundaries and central locations derived from the UAV LiDAR-based DEM with the results from the numerical terrain map, some discrepancies were observed. These differences can be attributed to the precision disparities between the two methods. Notably, some dolines exhibited significant differences in area and shape compared to the previous survey, indicating that UAV LiDAR technology is effective for the precise detection and recording of microtopographic features (see Figure 8).

### 4.3. Morphometric Analysis of Detected Dolines

Table 4 summarizes the key morphological indices of the seven dolines extracted from the UAV LiDAR-based DEM. For each doline, the major axis (*x*-axis), minor axis (*y*-axis), perimeter, area, and central elevation were calculated, providing insight into the morphological diversity and spatial characteristics of the dolines in the Gulneomjae region.

The length of the major axes of the dolines ranged from a minimum of 21.41 m to a maximum of 67.91 m, while the length of the minor axes ranged from a minimum of 24.40 m to a maximum of 157.86 m. Notably, Doline ID 2, with a major axis of 67.91 m and a minor axis of 157.86 m, was the largest, with a perimeter of 399.06 m and an area of 6867.14 m^2^, making it the largest among the entire set of analyzed dolines. In contrast, Doline ID 7 was the smallest, with an area of 423.64 m^2^ and a perimeter of 82.01 m.

The central elevation of the dolines generally ranged from 190 m to 215 m, with Doline ID 6 being the highest at an elevation of 215.27 m. Despite having a relatively large area (4492.82 m^2^), this doline’s central elevation indicates its position at a relatively higher point within the mountainous terrain.

These results indicate a high degree of topographic diversity among the dolines in terms of their dimensional characteristics and spatial distribution. Furthermore, the UAV LiDAR-based high-resolution DEM proves to be an effective tool for quantitatively capturing these subtle differences.

**Figure 9:**
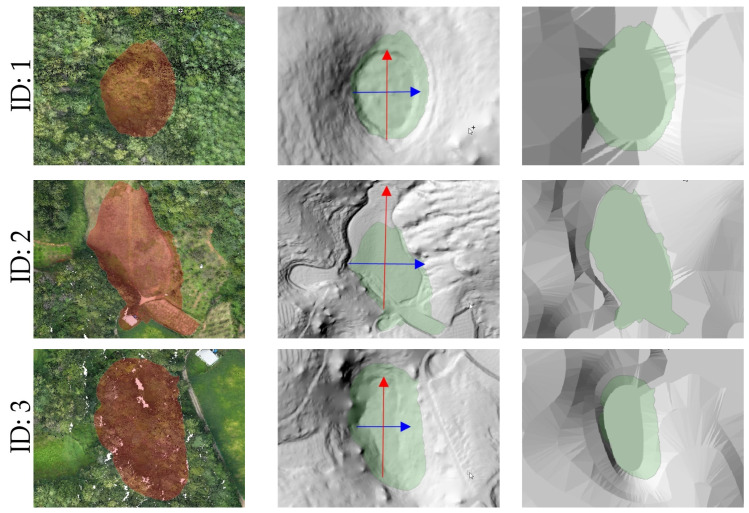

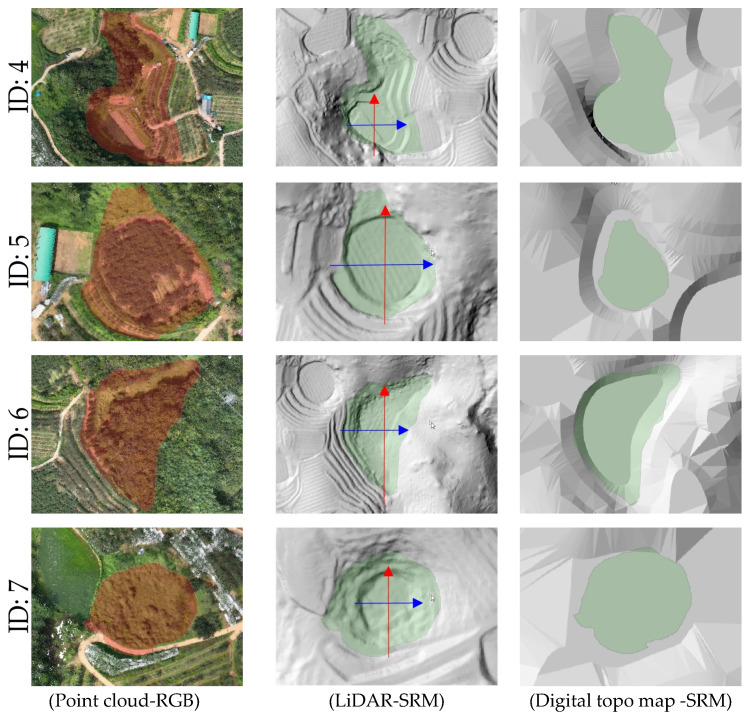
Comparison of RGB point cloud with doline boundaries (**left**), shaded relief map generated from UAV LiDAR (**center**), and shaded relief map based on the 1:5000-scale topographic map (**right**). (Blue line is *x*-axis, red line is *y*-axis, and the red and green polygons are the results of a survey conducted in 2013.)

**Figure 10:**
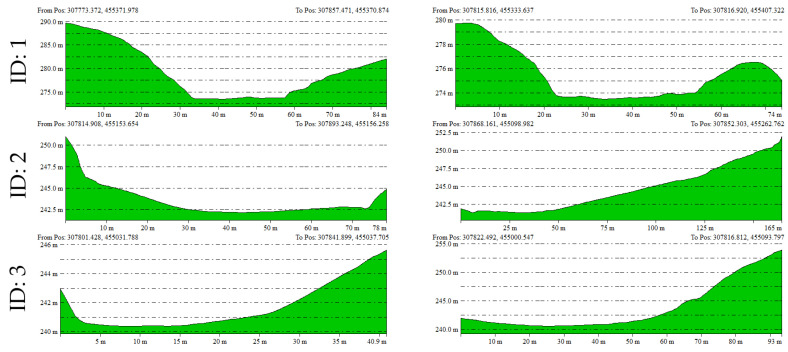

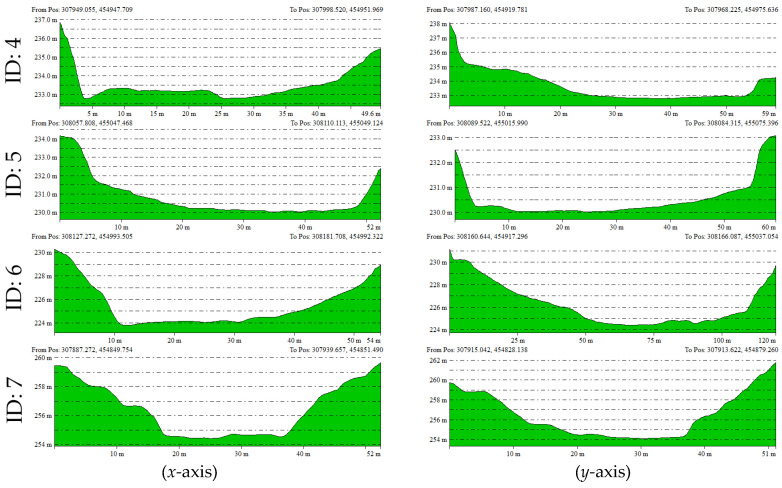
Elevation profiles along the *x*- and *y*-axes extracted from the UAV LiDAR-derived DEM for each doline.

Using the UAV LiDAR-based DEM, elevation profiles in both the X and Y axes were extracted for each doline and analyzed (Figure 9). The elevation profiles are useful for understanding the depth, slope morphology, and symmetry of each doline, allowing for a visual analysis of the developmental characteristics and subsidence structures of the terrain (Figure 10).

For example, Doline ID 1 and 7, although smaller in area, exhibited steep slope shapes, while Doline ID 2 and 6 showed relatively gentle and wide basin-shaped forms. These findings are likely reflective of the heterogeneous characteristics influenced by factors such as the formation period of the dolines, erosion intensity, and geological conditions.

## 5. Discussion

This study empirically demonstrated that UAV LiDAR technology is highly effective for the precise morphological analysis of karst mountain dolines. The DEM with a resolution of 0.25 m and a high point density (98 pts/m^2^) was able to capture fine topographic features, such as the boundaries, depth, and cross-sectional shapes of the dolines, much more clearly than the traditional 1:5000 scale topographic map-based analysis method. In particular, UAV LiDAR exhibited excellent ground surface detection capabilities, even in areas with dense vegetation or steep mountainous terrain. Compared to SfM-based UAV photogrammetry or aerial LiDAR, the UAV LiDAR method demonstrated higher point density and better ground penetration in vegetated areas, enabling more detailed surface modeling [1,5,38].

The vertical accuracy of the UAV LiDAR-derived DEM was assessed through RTK-GNSS positioning capabilities of the DJI Matrice 300 RTK system. As shown in Table 2, the IMU trajectory errors indicate positional RMSE values of 0.00607 m (*x*-axis), 0.00482 m (*y*-axis), and 0.00674 m (*z*-axis), with average positioning errors of 0.0068 m, 0.00617 m, and 0.00806 m for the *x*, *y*, and *z* axes, respectively. These values demonstrate sub-centimeter horizontal accuracy and approximately 8 mm vertical accuracy, which substantially exceeds the precision requirements for 0.25 m resolution DEM generation. However, we acknowledge that independent ground control point validation would provide additional verification of these accuracy claims. The RTK-GNSS system provides real-time corrections that minimize systematic errors, but future studies should incorporate independent checkpoints using high-precision survey-grade GNSS equipment to validate the absolute accuracy claims. The reported accuracy values represent the theoretical precision achievable under optimal conditions and may vary slightly across different terrain types and vegetation densities within the study area

The quantitative terrain analysis of the dolines revealed significant spatial heterogeneity and various subsidence patterns, which are consistent with the findings of [3,4]. For example, certain dolines exhibited steep subsidence structures, while others showed gentle and wide basin-like forms, reflecting differences in terrain formation processes and erosion conditions. These characteristics can serve as fundamental data for future doline classification and subsidence prediction model development.

The morphometric parameters reported in this study must be interpreted within the context of the 0.25 m DEM resolution. While our measurements report precise values (e.g., *x*-axis length of 25.130 m for Doline ID 1), the effective precision is inherently limited by the spatial resolution of the underlying data. For a 0.25 m resolution DEM, the realistic precision for linear measurements should be considered as ±0.25 m, and for area calculations, the uncertainty propagates to approximately ±1.0–2.0 m^2^ depending on the complexity of the doline boundary. The high point density (98 points/m^2^) enables accurate interpolation between grid points, potentially improving the effective precision beyond the nominal grid resolution. However, we acknowledge that the reported morphometric values should be interpreted as estimates with inherent uncertainties related to the DEM resolution. For critical applications requiring higher precision, finer-resolution DEMs (e.g., 0.1 m or 0.05 m) would be necessary, though this would require adjusted flight parameters and increased data processing requirements.

Additionally, the comparison with the 2013 topographic map-based survey results demonstrated that UAV LiDAR technology is an effective tool for detecting terrain changes and performing time-series analysis. The disappearance or modification of some dolines can be interpreted not merely as detection errors but as actual changes in the terrain (e.g., cultivation, erosion), suggesting that the repetitive measurement capabilities of UAV LiDAR are suitable for future terrain change monitoring [14,37]. However, the apparent disappearance or modification of two dolines (previously identified as 1–8 and 1–9 in the 2013 study) requires careful interpretation and explicit acknowledgment of uncertainty. The eleven-year temporal gap between datasets introduces significant uncertainty, as karst landscapes can undergo rapid changes, particularly in areas subjected to human activities. Additionally, the 2013 study’s reliance on field surveys combined with 1:5000-scale topographic maps may have different detection thresholds compared to our high-resolution LiDAR approach. Future research should incorporate systematic field validation and establish a more frequent monitoring protocol to distinguish between actual geomorphological changes and methodological artifacts.

In addition, this study also has several limitations. First, the surface filtering algorithm used in DEM generation is not perfect, so the influence of residual vegetation may still be slightly reflected in some flat areas. Second, an automatic doline extraction algorithm was not applied, and most of the analysis was conducted manually. The reported average LiDAR point density of 98 points/m^2^ requires explicit clarification to ensure accurate interpretation. This density represents the raw point cloud data collected during the UAV LiDAR survey, before ground filtering and classification processes. Following preprocessing and ground point extraction using DJI Terra software, the density of ground-classified points used for DEM generation was approximately 15–20 points/m^2^, depending on terrain characteristics and vegetation cover. Point density exhibited spatial variability across the study area. In open terrain with minimal vegetation, ground point density approached 90% of the raw collection rate (approximately 88 points/m^2^), while in densely vegetated areas, ground point density decreased to approximately 15–20% of raw collection (15–20 points/m^2^) due to vegetation filtering. Steep slope areas showed intermediate ground point densities of approximately 40–60 points/m^2^. These variations are typical for LiDAR surveys in mixed terrain and vegetation conditions. The high raw point density ensures adequate ground point coverage even after vegetation filtering, but users should be aware that effective ground point density varies spatially. This variability potentially affects the local accuracy of the derived DEM, with higher confidence in morphometric measurements in areas with higher ground point density. Future applications should consider this spatial variability when interpreting results and may benefit from reporting confidence intervals based on local point density variations. Future research should introduce LiDAR-based sinkhole automatic extraction algorithms, as seen in [4], or incorporate machine learning-based automatic classification methods.

Lastly, UAV LiDAR data has high potential for use not only in terrain analysis but also in ecological and environmental monitoring. For example, as seen in the studies by [11,24], integrating LiDAR data for biomass estimation and vegetation structure analysis could extend to tracking changes in the ecological environment of dolines. This study has shown that UAV LiDAR has the potential to evolve beyond a simple terrain surveying tool into a comprehensive environmental analysis platform.

To evaluate the effectiveness of the UAV LiDAR-derived DEM in identifying doline features, we compared our results with those from a 2013 field study that utilized a 1:5000-scale digital elevation model based on the National Basic Map. Although more recent official field surveys or high-resolution datasets were not available for the study area, the 2013 dataset was chosen as a benchmark due to its prior use in karst landform analysis within the region. We acknowledge that the ten-year temporal gap between datasets may introduce potential morphological changes resulting from natural processes such as subsidence, erosion, or vegetation growth. Nonetheless, this temporal comparison provides valuable insight into the stability or variability of doline features over time and further highlights the advantages of UAV LiDAR in capturing updated and detailed terrain information.

## 6. Conclusions

This study utilized UAV LiDAR technology to precisely analyze the fine topography of dolines in the karst mountainous region of Gulneomjae, Hogye-myeon, Mungyeong City, South Korea, an area characterized by dense vegetation and steep slopes. The precision and potential of this method were compared with traditional topographic map-based methods. From this study, the following major conclusions were drawn:

First, the UAV LiDAR-based DEM provided higher precision in terms of resolution (0.25 m) and point density (98 pts/m^2^) compared to the 1:5000 scale topographic map-based DEM. Although both DEMs enabled doline detection, the higher spatial resolution and point density of the UAV LiDAR data facilitated the extraction of subtle topographic features such as slope curvature and asymmetry, enhancing the reliability of morphometric classification. This enabled more accurate identification of the boundaries and internal structures of dolines located beneath dense vegetation, demonstrating the potential of UAV LiDAR as an alternative technology to overcome the limitations of traditional methods.

Second, in comparison with the 2013 topographic map-based study, some dolines (e.g., 1–8, 1–9) were not identified in the current UAV LiDAR data. This is interpreted as being due to terrain changes, such as cultivation, or limitations in the resolution of the previous data. This suggests that UAV LiDAR technology is an effective tool for detecting terrain changes and providing more precise detection compared to past methods.

Third, the morphological analysis, based on quantitative indicators such as the perimeter, area, and depth of the dolines, can serve as valuable foundational data for the development of automatic doline classification algorithms in the future. In particular, cross-sections and shaded relief maps generated based on high-resolution DEM have high potential as training data for numerical analysis and machine learning-based models.

Fourth, UAV LiDAR can reliably acquire high-precision terrain data even in areas that are difficult for personnel to access, and it can be applied to various spatial analysis studies in the future, including terrain stability assessment in forests, subsidence monitoring, and natural disaster prediction. Given its capability for repeated surveys, it also has great potential for time-series-based terrain change analysis.

Fifth, UAV LiDAR data goes beyond simple terrain surveying and can be expanded to machine learning and artificial intelligence-based analysis. When classification, anomaly detection, and change detection models using large-scale point clouds are applied, it can evolve into a system for predicting terrain changes and automatic detection. This can contribute to the automation of terrain management and improvement in prediction accuracy.

Future research should focus on developing methodologies that automate terrain classification, erosion and sedimentation prediction, and environmental change monitoring using artificial intelligence-based pattern recognition technologies with accumulated LiDAR and image data. Additionally, comparing and analyzing past and present terrain data to learn specific change patterns and building models to simulate future terrain changes will be crucial research tasks.

## Figures and Tables

**Table 1:** Flight configuration for study site.

UAV Flight Plan		LiDAR Payload Setting	
Date	23 September 2024	Reflect mode	Penta
Flight altitude	150 m	Sampling rate	240 kHz
Side overlap (RGB)	61%	Scan mode	Repeated
Front overlap (RGB)	70%	Side overlap	50%
Area covered	0.64 km^2^	Mode	Interval
Average GSD	4.03 cm/pix	Density	98 pts/m^2^

**Table 2:** Accuracy parameter (IMU Trajectory Error).

Parameter	X (E) RMSE	Average X (E)	Y (N) RMSE	Average Y (N)	Z (U) RMSE	Average Z (U)
Location	0.00607 m	0.0068 m	0.00482 m	0.00617 m	0.00674 m	0.00806 m
Attitude	0.0000019 rad	0.0000641 rad	0.0000044 rad	0.0000649 rad	0.000026 rad	0.0002406 rad

**Table 3:** Output Parameter (Point Cloud Density).

Scale	Point Cloud Average Density	Point Cloud Standard Density	Grid Side Length	Total Grid Number	Non-Conforming Grid Ratio
1:500	90 points/m^2^	16 points/m^2^	0.25 m	2,216,644	9.07%
1:1000	90 points/m^2^	4 points/m^2^	0.5 m	581,296	4.67%
1:2000	90 points/m^2^	1 points/m^2^	1.0 m	149,952	3.09%

**Table 4:** Morphometric characteristics of the seven dolines derived from UAV LiDAR DEMs.

ID	*X*-Axis Length (m)	*Y*-Axis Length (m)	Perimeter (m)	Area (m^2^)	Elevation (m)
1	25.130	33.964	93.012	662.721	201.624
2	67.907	157.857	399.061	6867.140	194.522
3	25.307	66.473	177.999	1030.439	199.624
4	41.523	50.807	156.387	1627.121	190.939
5	44.233	52.098	153.546	1784.863	190.258
6	40.764	112.664	351.753	4492.820	215.273
7	21.405	24.399	82.011	423.643	195.165

## Data Availability

Data is available from the corresponding author upon reasonable request.
